# Polarization-Modulated, Goos–Hanchen Shift Sensing for Common Mode Drift Suppression

**DOI:** 10.3390/s19092088

**Published:** 2019-05-05

**Authors:** Yuhang Wan, Mengxuan Cheng, Zheng Zheng, Kai Liu

**Affiliations:** 1 School of Electronics and Information Engineering, Beihang University, 37 Xueyuan Rd., Beijing 100083, China; yuhangwan@buaa.edu.cn (Y.W.); cmx_325@buaa.edu.cn (M.C.); zhengzheng@buaa.edu.cn (Z.Z.); 2 Collaborative Innovation Center of Geospatial Technology, 129 Luoyu Rd., Wuhan 430079, China; 3 Beijing Advanced Innovation Center for Big Date-based Precision Medicine, Beihang University, 37 Xueyuan Rd., Beijing 100083, China

**Keywords:** optical instruments, surface waves, optics at surfaces, refractive index sensors

## Abstract

A polarization-modulation-based Goos–Hanchen (GH) sensing scheme leveraging the polarization-dependence of the Bloch surface wave enhanced GH shift is proposed and experimentally demonstrated. Based on a simple setup utilizing a liquid crystal modulator to switch the polarization state of the input beam periodically, the alternating positions of the reflected beam for both polarizations are monitored by a lock-in amplifier to handily retrieve the GH shift signal. The conventional direct measurement of the beam position for the target state of polarization is vulnerable to instabilities in the optomechanical setup and alignment. Our proposed scheme provides a sensitive yet robust GH shift-sensing setup where the common mode drift and noise could be suppressed to ensure better system stability.

## 1. Introduction

For a couple of decades, surface plasmon resonance (SPR) has been extensively studied as a powerful diagnostic tool for real-time, label-free bio-related detection [1]. This kind of surface wave-based technology has been successfully accepted as the gold standard for molecular interaction analysis. As time goes by, achieving ever greater sensitivity is on demand and has become the goal of many research efforts for similar technologies. Therefore, different interrogation schemes and sensing methods of SPR have been thoroughly investigated, and a lot of efforts have been spent for reaching higher sensitivity detection. It has been discussed that as the phase change is more abrupt than amplitude change, an interrogation method based on phase detection might be more sensitive than one based on intensity detection [2]. Therefore, phase detection methods have been studied [3,4]. However, in general, the phase cannot be measured directly, and it needs to be retrieved from the intensity of the interference pattern, where complicated, bulky interferometric setups usually need to be built. In order to simplify the interferometric setups for phase detection, phase-related or phase-enhanced intensity measurement has been studied to improve the sensitivity [5]. Another scheme is to directly measure the Goos–Hanchen (GH) shift which is proportional to the phase change induced by the SPR effect, where a potentially simple yet sensitive sensing system for biosensing applications has been proposed and demonstrated [6].

The GH effect is an interesting physical optical phenomenon that occurs during the nonspecular reflection, where the position of the reflected beam deviates from geometric optic prediction with a lateral displacement [7]. It could be visualized as the wave propagating along the surface for a while before being reflected, and the magnitude of this shift is often related to the penetration depth of the surface wave at the interface. The GH effect has attracted a lot of attention since it was discovered for the profound physical meaning behind it, and different models have been proposed for the explanation. For a bounded incident plane wave, the GH shift could be estimated by the derivative of the phase jump [8]; while for a more general incident beam with different wave vectors, the derivatives of the phase changes for different wave vectors will be summed up and taken into consideration for the GH effect [9]. For a long time, the GH effect has been mainly studied theoretically, for the magnitude of the lateral shift is at the order of the wavelength, which is so minute for the light wave that it could be hardly observed in a system outside of the laboratory. Therefore, studies have been carried out to enhance the GH effect for the potential practical application, among which it was discovered that the excitation of the surface wave would be an efficient way for GH effect enhancement due to the associated significant phase jump [6,9,10,11]. For the SPR effect, the phase change is usually more significant than the intensity; therefore, observing the phase change has been regarded to be able to get better sensitivity than the intensity. The corresponding GH shift is shown to be larger than tens of microns [6,9,12]. This amount of beam shift could be measured directly in experiments utilizing a simple setup, compared to complicated interferometry setups for phase measurement. Yet, due to the characteristics of the surface plasmon wave, the maximal GH shift is no more than 100 times of wavelength.

In contrast to SPR, another type of surface electromagnetic waves, Bloch surface wave (BSW) can be excited at the interface of a truncated one-dimensional photonic crystal (PC) slab and shares certain similarities to the surface plasmon wave [13]. As it propagates along the interface between two dielectrics, a much lower optical loss is obtained for the BSW, resulting in a longer lateral propagation distance and penetration depth compared to SPR. Lots of efforts have been carried out demonstrating the long propagation distance [14,15,16], and utilizing the BSW resonance for applications like sensing [16,17]. In addition to its low propagation loss due to the absence of Ohmic loss in the structure, its flexibility in design offers a wide range of choices in the structure parameters and the corresponding optical properties. The design and optimization of the band gap structure have been discussed for the BSW excitation [18]. It has been demonstrated that with proper design, a giant GH effect beyond hundreds of microns level, even into the submillimeter range, could be observed experimentally for the excitation of the BSW [10,19,20]. As the BSW is highly affected by the characteristic of the material adjacent to the interface surface, the BSW-induced GH shift could be manipulated through the refractive index of the adjacent medium. With this significantly enhanced GH shift, practical applications like switching [21] and sensing [22] would be possible. 

In such a BSW-induced GH shift sensing system, the position of the reflected beam is measured using a position-sensitive detector (PSD) or an image sensor like charge-coupled device (CCD). Compared to conventional surface wave-based sensing systems where the optical power is monitored, the stability of the position measurement directly affects the system performance of the GH shift sensing system. Yet, in real-world systems, the inevitable changes in instrument temperature, vibration of the mechanical parts, and more would contribute to the results read from the PSD, which would greatly affect the capability of a GH shift sensing system to realize high-sensitivity detection. Therefore, it is of critical importance to reduce or eliminate such drift and instability in a GH shift sensing setup. 

As is known, the excitation of the surface wave that could enhance the GH shift, including SPW and BSW, depends strongly on the polarization. It is noted that a BSW structure can be designed to possess highly different GH responses for different states of polarization. Based on this, we propose and experimentally demonstrate a polarization-modulated GH sensing scheme using a BSW enhanced GH shift sensor. By measuring the alternating position of the reflected beam with and without the GH response, the retrieved GH shift is free from the common mode noise and drift compared to the conventional direct position measuring systems. Therefore, a sensitive yet robust GH sensing system could be realized with a simple setup.

## 2. Proposed Scheme and Experimental Setup

**Figure 1:**
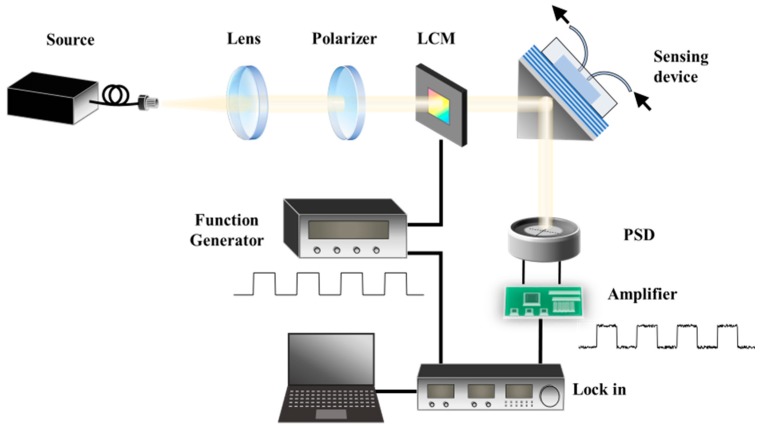
Schematic diagram of the proposed scheme.

The schematic diagram of the proposed sensing scheme is shown in Figure 1, where the GH shift sensor chip is a specially designed one-dimensional PC slab terminated by a buffer layer, where a flow-cell made of Polydimethylsiloxane (PDMS) is attached. The experimental setup is based on the conventional Kretschmann configuration for the excitation of the surface wave. The sensor chip is attached to the coupling prism that is fixed at an incident angle where the BSW can be excited.

**Figure 2:**
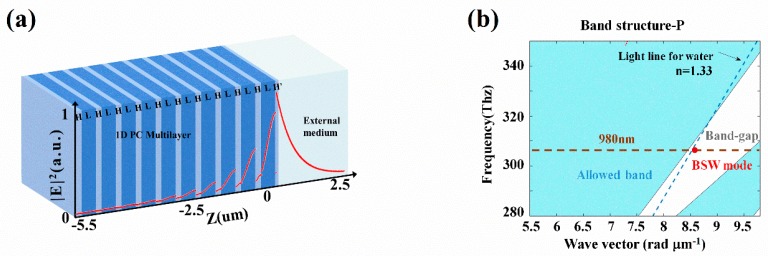
(**a**) Schematic of the BSW enhanced GH shift sensor and the normalized electric field distribution of the excited BSW; (**b**) simulated band structure of an ideally infinite 1D PC structure for P-polarization and the BSW mode excited (red circle). The light blue region is the allowed band and the white region is the band-gap. Dash line is the light line for water (*n* = 1.33).

**Figure 3:**
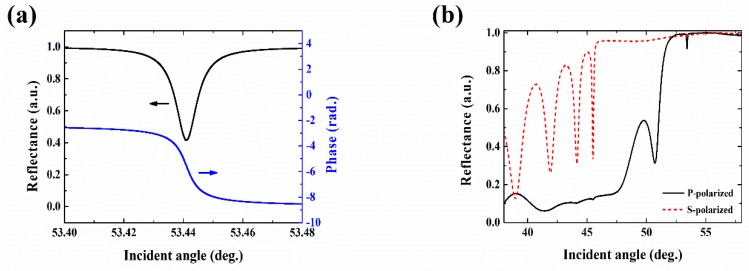
(**a**) Simulated angular reflectance and phase response when the external medium is water for P-polarized input. (**b**) Measured angular reflectance when the external medium is water for P-polarized and S-polarized input.

The structure of the BSW enhanced GH shift sensor chip is sketched in Figure 2a, which can be expressed as substrate /(HL)^10^H’/cladding. For P-polarized incident light at the wavelength of 980 nm, the device is designed to be able to excite the BSW mode with water as the cladding (*n* = 1.33). The substrate is made up of ZF10 glass (*n*_S_ = 1.668), with TiO_2_ and SiO_2_ evaporated alternatively as high index layer (H, *n*_H_ = 2.30) and low index layer (L, *n*_L_ = 1.434), respectively, where the buffer layer adjacent to the external medium is also TiO_2_ (H’) with a different thickness. The thicknesses of the high index layer, the low index layer, and the buffer layer are 163 nm, 391 nm, and 23 nm, respectively. In order to represent the intrinsic loss of the material and the scattering loss from the interfacial roughness induced through the fabrication process, an extinction coefficient of 2 × 10^−4^ is added to the imaginary part for the refractive index of the TiO_2_ for calculation to match the experimental observation [10]. Figure 2b illustrates the band structure of an ideal periodical, infinite photonic crystal for P polarization calculated through the transfer matrix method [23], where the excited BSW mode is marked with a red circle. It shows that for the incident wavelength, the light line for water (*n* = 1.33) is just beyond the rising edge of the forbidden band, and the excited BSW mode sits in the forbidden band and lies closely to the light line for water, which means that a slight refractive index change in water would strongly affect the excitation of the BSW, i.e., the design is suitable for sensing in an aqueous solution environment. The excitation of the BSW mode could be observed from reflectance simulation using Fresnel equations, as shown in Figure 3a, where a sharp attenuation dip beyond the total internal reflection is plotted with a corresponding abrupt phase jump for comparison. As the structure is designed for P polarization, it has been confirmed that for the same cladding with an S polarized input, no surface wave will be excited.

In the experimental setup as shown in Figure 1, a fiber-coupled laser emitting at 980 nm was used as the light source, where the output was collimated from the fiber endface and then followed by a polarizer to illuminate on the said BSW enhanced GH shift sensor chip. The reflected beam was then measured by a position-sensitive detector (PSD, S3979, Hamamatsu, Japan) with a PSD signal amplifier (OT-301, On-Trak Photonics, Irvine, CA, USA). A liquid crystal spatial light modulator (LCM, CRi 128D, Meadowlark Optics, Frederick, CO, USA) was placed after the polarizer in the optical path, to control the state of polarization of the light beam illuminating onto the sensor chip. The LCM is software-triggered by a computer, which also generates a synchronization signal for the lock-in, to continuously switch the polarization state between P polarization and S polarization. As is explained before, if the input beam to the sensor is P-polarized, the excitation of the BSW will greatly enhance the GH effect, resulting in a lateral shift of the reflected beam corresponding to the refractive index change of the cladding medium, and when the input to the sensor is S-polarized, no BSW is excited and no GH shift exists as well. Therefore, the PSD’s signal can serve as the reference under the S-polarization state. The modulated output read from the PSD amplifier is sent into a lock-in amplifier (SR830, Stanford Research Systems, Sunnyvale, CA, USA). During the experiment, the incident angle of the light to the sensor chip was fixed, and test samples were aqueous solutions of glycerol with various concentrations. Samples were injected into the flowcell of the sensor through a homemade microfluidic control system.

## 3. Results and Discussions

**Figure 4:**
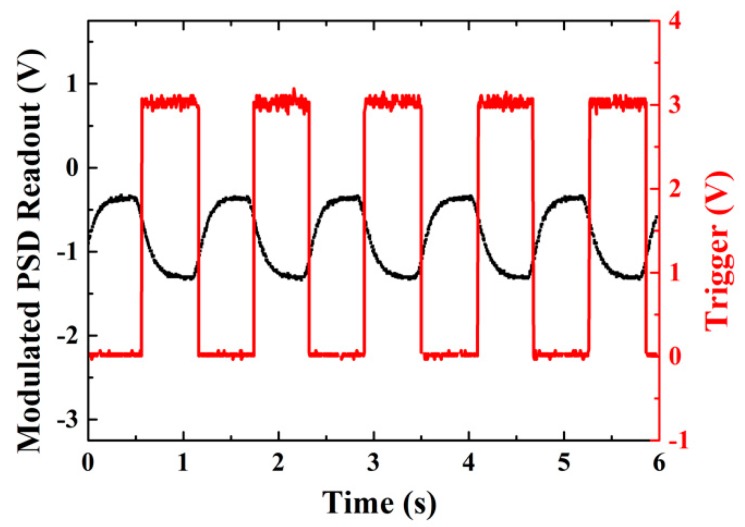
Polarization modulated position-sensitive detector (PSD) readout and synchronized trigger.

**Figure 5:**
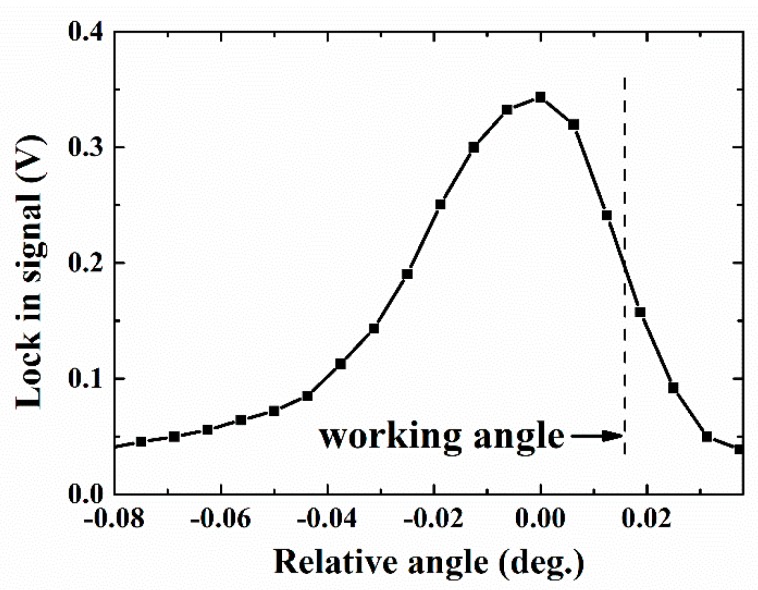
The measured Goos–Hanchen (GH) shift readout from the lock-in amplifier with the Bloch surface wave (BSW) excitation for water cladding (dark dash line: The working angle).

At the beginning of the experiment, it is important to confirm the excitation of the BSW, which could be observed by angular scanning the reflected intensity [17], or the phase-related Goos-Hanchen effect [22], as previous studies have shown. The measured angular reflectance of P polarization with water as cladded medium is plotted as well as that of S polarization in Figure 3b. Figure 4 shows the polarization modulated signal measured by the PSD, with the high and low representing the position of the reflected beam for P and S polarized input, respectively. The modulation frequency is set at 0.86 Hz. This is limited by the relatively slow switching speed of the LCM, which is clearly seen as the relatively long rise and fall time (~200 ms) of the observed PSD signal. The corresponding synchronized signal of the LCM is plotted as well. By sending the polarization-modulated PSD signal into the lock-in amplifier, the difference between the beam positions of the P polarization and the S polarization, i.e., the GH shift could be retrieved. The angular scanning of GH shift variation around the BSW excitation is measured from the polarization modulated PSD signal using the lock-in amplifier, as shown in Figure 5. During the sensing experiment, the working angle is fixed around the position where the BSW is excited and the GH shift is most sensitive to the external medium refractive index change. Then, the measured GH shift change is proportional to the refractive index change of the external medium.

**Figure 6:**
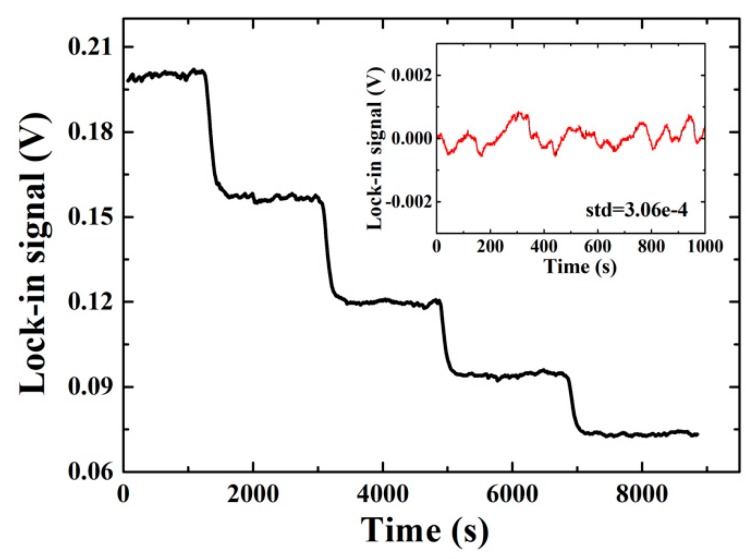
Real-time record of the lock-in signal with different samples.

The real-time record of the retrieved GH shifts from the polarization-modulated PSD signal measured through the lock-in amplifier for different injections is shown in Figure 6, where glycerol solutions with different concentrations (0.1%–0.5% in wt, the equiv. index difference = 1.17 × 10^−4^ RIU [24]) were used as test samples. Figure 6 is the typical real-time monitoring of the PSD signals, where each step of the signal represents a concentration change. From Figure 6, it shows that by retrieving the GH shift from the polarization modulated PSD signal, the common mode drift could be suppressed efficiently, resulting in a stable GH shift for each step, despite the relatively very long measurement time. The root-mean-square (RMS) value of the signal fluctuation is calculated to be ~3.1 × 10^−4^ V without much effort for environment control and vibration suppression of the setup.

For the previous direct position measurement-based sensing schemes [6,22], though the working position is fixed during the experiment, the measured signal taken as the GH shift is usually a combination of the actual GH shift caused by the BSW excitation and a position change of the reflected beam, which usually varies with the incident angle linearly. In that case, the incident angle drift due to the instability of the optical mounts might cause fluctuations to both parts. In the current scheme, although the potential drift of the incident angle due to the instability of the mounts might still cause fluctuations of the GH shift, especially around the resonance where the angular sensitivity is approaching the maximum, the latter part, i.e., the position change of the reflected beam varying with the incident angle could be removed automatically by alternatively collecting both the signal and the reference through two polarizations. In the proposed setup, the GH shift retrieved from the polarization modulation is independent from the absolute position of the reflected beam, where the common mode noise and drift introduced by the movement of the experimental setup could be suppressed. Further, the proposed method shows good tolerance for the alignment of the experimental setup, which makes setting up such a system much easier. On the other hand, by reading the signal through the lock-in amplifier, a minute position change could be obtained with better signal-to-noise ratio than direct position measurements.

We note that the current modulation frequency is only in the order of ~1 Hz, and the switching speed is limited by the specific LCM device that happens to be available to this experiment. Under the current limitation, the rising/falling time of the signal takes near half of the high/low period, which might reduce the retrieved GH shift signal by average. Further, the limited modulation rate prevents us from removing more environmental disturbance with higher frequency components from the signal. However, LCMs with much faster modulation speed (for example, the video rate) have become widely available, and it is noted that the response time for liquid crystal modulators could be shortened to submillisecond [25]. In that case, a modulation speed up to several kHz is possible. By increasing the modulation frequency, the noise, especially the higher-frequency noise, could be better suppressed, and much improved results could be expected.

## 4. Conclusions

A polarization-modulated, GH shift sensing system for common mode noise and drift suppression is proposed and experimentally demonstrated for a Bloch surface wave sensor. By switching the polarization state of the input light between S and P, the position of the reflected beam under the influence of the GH shift effect was recorded for both polarizations periodically. A lock-in amplifier can be utilized to conveniently retrieve the GH shift information by extracting the modulation depth of the reflected beam position signal. Compared to the previous GH sensing schemes, the common mode noise and drift introduced by the mechanical instability of the opto-mechanical setup could be removed easily. Therefore, by utilizing a liquid crystal modulator, a sensitive yet robust GH sensing system is presented with a simple and compact configuration. By introducing spatial light modulators with higher modulation speed, the overall performance of our scheme could be further improved by suppressing more noise at higher frequencies.
